# High-Density Energetic Metal–Organic Frameworks Based on the 5,5′-Dinitro-2*H*,2′*H*-3,3′-bi-1,2,4-triazole

**DOI:** 10.3390/molecules22071068

**Published:** 2017-06-26

**Authors:** Yalu Dong, Panpan Peng, Baoping Hu, Hui Su, Shenghua Li, Siping Pang

**Affiliations:** School of Materials Science & Engineering, Beijing Institute of Technology, Beijing 100081, China; mrmichelle@163.com (Y.D.); double_pan_peng@163.com (P.P.); m18811301152@163.com (B.H.); a178541@126.com (H.S.)

**Keywords:** energetic materials, metal organic frameworks, densities, polynitro heterocyclic ligands

## Abstract

High-energy metal–organic frameworks (MOFs) based on nitrogen-rich ligands are an emerging class of explosives, and density is one of the positive factors that can influence the performance of energetic materials. Thus, it is important to design and synthesize high-density energetic MOFs. In the present work, hydrothermal reactions of Cu(II) with the rigid polynitro heterocyclic ligands 5,5′-dinitro-2*H*,2′*H*-3,3′-bi-1,2,4-triazole (DNBT) and 5,5′-dinitro-3,3′-bis-1,2,4-triazole-1-diol (DNBTO) gave two high-density MOFs: [Cu(DNBT)(ATRZ)_3_]_n_ (**1**) and [Cu(DNBTO)(ATRZ)_2_(H_2_O)_2_]_n_ (**2**), where ATRZ represents 4,4′-azo-1,2,4-triazole. The structures were characterized by infrared spectroscopy, elemental analysis, ultraviolet-visible (UV) absorption spectroscopy and single-crystal X-ray diffraction. Their thermal stabilities were also determined by thermogravimetric/differential scanning calorimetry analysis (TG/DSC). The results revealed that complex **1** has a two-dimensional porous framework that possesses the most stable chair conformations (like cyclohexane), whereas complex **2** has a one-dimensional polymeric structure. Compared with previously reported MOFs based on copper ions, the complexes have higher density (ρ = 1.93 g cm^−3^ for complex **1** and ρ = 1.96 g cm^−3^ for complex **2**) and high thermal stability (decomposition temperatures of 323 °C for complex **1** and 333.3 °C for complex **2**), especially because of the introduction of an N–O bond in complex **2**. We anticipate that these two complexes would be potential high-energy density materials.

## 1. Introduction

High-energy density materials (HEDMs) are widely used in explosives, propellants, and pyrotechnics [1,2,3,4,5,6,7,8]. HEDMs play a significant role in military and civilian applications, which has aroused the extensive interest of chemists, such as Klapötke [2,3,4] and Shreeve [5,6,7,8]. A large number of energetic materials have been synthesized with nitrogen-rich heterocyclic compounds as precursors, such as dihydroxylammonium 5,5′-bistetrazole-1,1′-diolate (TKX-50) [9], ammonium 4-amino-3,5-dinitropyrazolate (LLM-116) [10], and 5,5′-dinitro-2*H*,2′*H*-3,3′-bi-1,2,4-triazole (DNBT) [11], and there has been a large increase in the density and detonation properties. In particular, as an emerging type of HEDMs, high-energy coordination polymers or metal–organic frameworks (MOFs) show potential as next-generation energetic materials, and the densities and thermal stabilities can also be tuned based on the character of the metal ions and organic linkers [12,13]. Because of their high formation enthalpies and greater number of coordination bonds, nitrogen-rich heterocyclic compounds as the organic linkers incorporated into MOFs, those types of MOFs have been studied frequently [14,15,16,17,18,19,20,21,22,23]. However, owing to the fact that most energetic ligands are parent heterocyclic or bi-heterocyclic molecules, such as 3, 3′-bi-1, 2, 4-triazole (BTRZ) [24] and 4, 4′-azo-1, 2, 4-triazole (ATRZ) [20], the densities of most energetic MOFs are on the low side. Therefore, the development of high-density energetic MOFs is a sought-after goal for extending the scope of their applications. In traditional energetic molecules, their densities and thermal stabilities can be easily improved by introducing functional groups, such as nitro groups (NO_2_), N–O bonds, and nitramino groups (NHNO_2_), but this type of formation is rarely seen in MOFs. To further improve the density, oxygen balance, nitrogen content, and detonation performances of energetic MOFs, polynitro heterocyclic compounds can be considered as ideal energetic ligands. However, there were few reports about high-energy MOFs with ligands based on polynitro heterocyclic compounds. Recently, Matzger reported an energetic MOF [MOF(Cu–DNBT)] based on a polynitro heterocyclic compound DNBT as a ligand [25], where the insensitivity to external stimuli and thermal stability (its decomposition temperature is above 300 °C) are promoted by formation of a structural framework. This proves that MOFs with polynitro heterocyclic compounds as ligands show potential as energetic materials. To further expand the structural framework (skeleton) and improve their energetic properties, we envision that nitro groups and N–O bonds could be introduced into MOFs.

**Figure 1:**
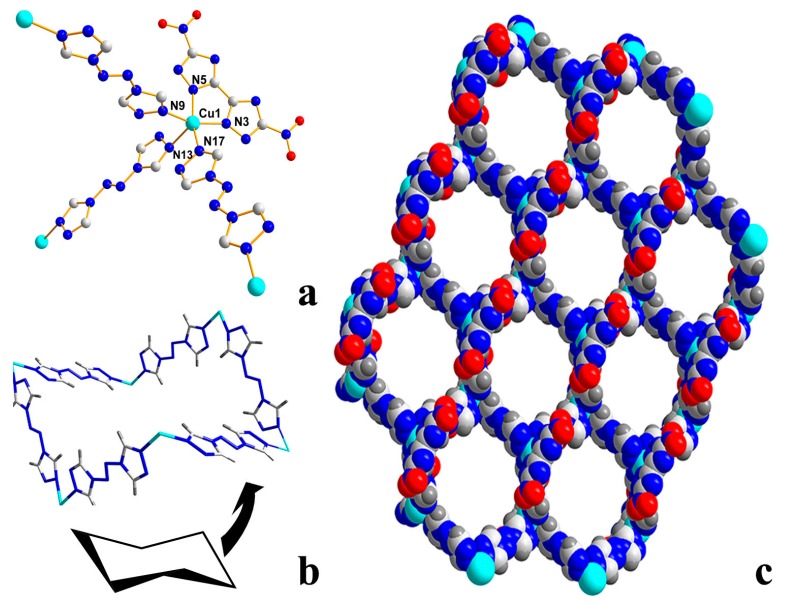
(**a**) Coordination environment of Cu(II) in **1**; (**b**) 2D chain in **1**; (**c**) 3D network in **1** by π-π interactions.

In this study, DNBT and its oxide, 5,5′-dinitro-3,3′-bis-1,2,4-triazole-1-diol (DNBTO), were used as ligands to assemble MOFs because of the following advantages: (1) these ligands possess high densities (e.g., DNBT 1.90 g cm^−3^) [11], high nitrogen contents (DNBT 49.5%, DNBTO 46.3%), and high heats of formation due to containing many high-energy N–N bonds (160 kJ mol^−1^) and N=N bonds (418 kJ mol^−1^) [26,27]; and (2) DNBT and DNBTO have different coordination modes, such as multidentate and building block bridging, as shown in Figure 1, offering the possibility for constructing unpredictable and fascinating MOFs. Cu(II) ions as the central atoms not only have good coordination ability with the N and O atoms of ligands, but they are also environmentally friendly ions compared with heavy metal ions such as lead [21,28] and mercury [16]. From the above considerations, the two novel energetic MOFs [Cu(DNBT)(ATRZ)_3_]_n_ (**1**) and [Cu(DNBTO)(ATRZ)_2_(H_2_O)_2_]_n_ (**2**) were prepared by the hydrothermal method and were characterized in detail by infrared spectroscopy, elemental analysis, ultraviolet-visible (UV) absorption spectroscopy and single-crystal X-ray diffraction. In addition, their thermal stabilities were determined by thermogravimetric/differential scanning calorimetry analysis (TG/DSC). The results revealed that the complexes **1** and **2** possess high densities (ρ = 1.93 g cm^−3^ for complex **1** and ρ = 1.96 g cm^−3^ for complex **2**) and high thermal stabilities (decomposition temperatures of 323.0 °C for complex **1** and 333.3 °C for complex **2**), especially because of introduction of an N–O bond in complex **2**.

## 2. Results and Discussion

### 2.1. Synthesis of Energetic Complexes

The copper complexes were synthesized by a simple one-step hydrothermal reaction of copper dinitrate pentahydrate [Cu(NO_3_)_2_·5H_2_O] with polynitro heterocyclic compounds (DNBT and DNBTO) and ATRZ in water. Complexes **1** and **2** are air stable, maintain their crystallinity for at least several weeks, and are insoluble in common organic solvents, such as dimethyl sulfoxide (DMSO), chloroform, methanol, ethanol, and acetone. The IR spectrum of complex **1** showed a strong band associated with the NO_2_ group (1540 cm^−1^), while complex **2** also had a strong band according to the N-O bond (1465 cm^−1^) in its IR spectrum (Figure S2, See the Supporting Information). The results showed the two polynitro ligands were successfully involved in their MOFs. To better characterize the structures, single-crystal X-ray experiments were performed. The experimental details for structural determination of the compounds are summarized in Table S1, and selected bond lengths and angles are given in Tables S2 and S3. The hydrogen bonding parameters are listed in Table S4 (See the Supporting Information). The results of XRD analysis are shown in Figure S3. Further information about the crystal structure determination is provided in the Supporting Information.

### 2.2. X-ray Crystallography

Complex **1** crystallizes in the triclinic space group P(-1) (see Table S1) with a 2D porous network, in which there is one crystallographically-independent copper atom. The molecular structure is shown in Figure 1a. DNBT adopts a bidentate bridging mode to coordinate to the copper atom. The asymmetry unit is made of one Cu(II) ion, one DNBT ligand, and three ATRZ ligands. The Cu(II) ion is penta-coordinated to two nitrogen atoms from DNBT ligands (N3 and N5) and three nitrogen atoms from ATRZ ligands (N9, N13 and N17). (Cu1-N3 = 2.006(3), Cu1-N5 = 2.006(9), Cu1-N9 = 1.991(5), Cu1-N13 = 2.249(1), and Cu1-N17 = 2.030(1) Å) and the N-Cu-N bond angles are in the range 79.29–159.52° (Table S2) in a distorted square pyramid. Figure 1b shows the 2D layer of complex **1**, in which adjacent Cu(II) centers are bridged by ATRZ ligands in three different directions to from a nearly hexagonal grid. In addition, the π-π interactions of triazole rings that results in molecular stacking planes, forming the 2D porous structure. The pore structure in the framework takes the most stable chair conformation (like cyclohexane).The presence of three unique molecular stacking plane orientations results in mixed molecular stacking (Figure 1c), which prevents interlayer sliding within the crystal lattice.

**Figure 2:**
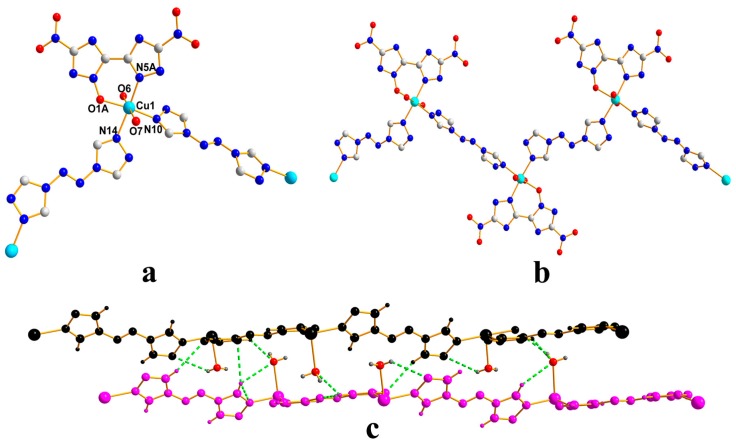
(**a**) Coordination environment of Cu(II) in **2**; (**b**) 1D chain in **2**; (**c**) 2D network in **2** formed by hydrogen-bonding interactions.

Complex **2** crystallizes in the monoclinic space group P2_1_/n (see Table S1) with a 1D porous MOF, in which there is one crystallographically-independent copper atom. The molecular structure is shown in Figure 2a. The asymmetry unit is composed of a copper ion as the center of the equatorial plane, and it includes one Cu(II) ion, one DNBTO ligand, two ATRZ ligands, and two H_2_O molecules (Figure 2). DNBTO adopts a bidentate bridging mode to coordinate the copper atom. The Cu(II) ion is hexa-coordinated to three nitrogen atoms from DNBTO and ATRZ ligands (N5, N10, and N14) and three oxygen atoms (O1, O6, and O7) from DNBTO and water molecules [Cu1–N5A = 1.954(9), Cu1–N10 = 2.004(4), Cu1–N14 = 2.016(0), Cu1–O1A = 1.965(6), Cu1–O6 = 2.326(5), and Cu1–O7 = 2.380(3) Å], and the N–Cu–N bond angles are in the range 81.95–174.03° (Table S3) in a distorted octahedron-like structure. ATRZ bridges the skeleton. The intermolecular hydrogen bonds between hydroxy and nitro groups are shown by dashed lines in Figure 2c. The hydrogen bonds and π-π interactions result in molecular stacking planes with an interplanar distance of 6.02 Å. Abundant hydrogen bonds, including O6–H6···N10, C5–H5···N11, C8–H8···O7, and C8–H8···O1A with distances range from 1.913 to 2.416 Å (see Table S4), between expanded chains result in a stable 1D structure.

### 2.3. Ultraviolet-Visible (UV) Absorption

**Figure 3:**
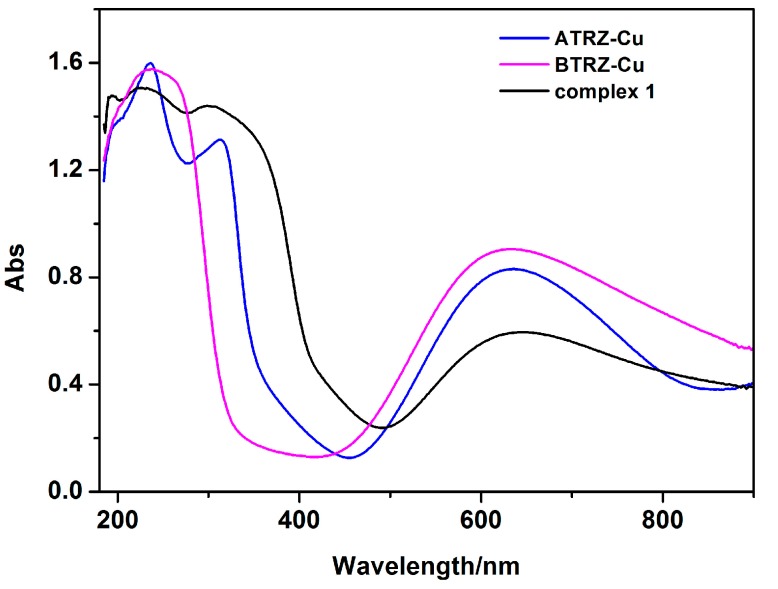
Ultraviolet absorption spectra of complex **1**, ATRZ-Cu and BTRZ-Cu.

The solid-state ultraviolet absorption spectra of three complexes [(ATRZ-Cu), (BTRZ-Cu), and **1**] at room temperature are depicted in Figure 3. By means of the ultraviolet absorption spectra of three complexes, the impact of nitro groups on the skeleton of MOFs based on nitrogen-rich heterocyclic ligands was investigated.

As can be seen from Figure 3, the three kinds of MOFs show strong absorption within the range 200–700 nm. In contrast to **1** and BTRZ-Cu, there is an additional strong peak in **1** at 310 nm, which is possibly attributable to the absorption of ATRZ. In addition, the absorption band of complex **1** at 200–400 nm becomes significantly broader, probably because of the strong π-π interactions between the adjacent triazole rings of the layers in **1,** which could reduce the π-π* transition energy. Meanwhile, the electron-withdrawing effect of the nitro group brought about the hypochromic effect of complex **1**.

### 2.4. Stability and Detonation Properties

**Figure 4:**
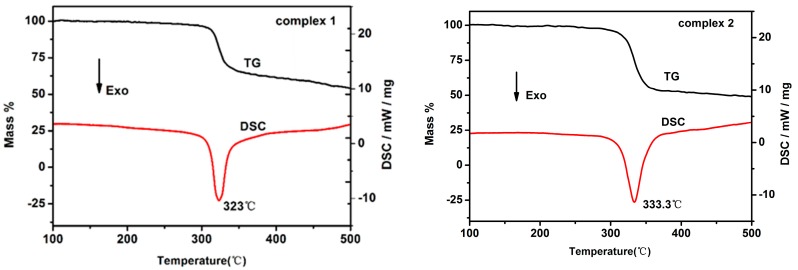
TG/DSC curves of complexes **1** and **2.**

The thermal decomposition temperatures of the complexes were determined by thermogravimetric/differential scanning calorimetry (TG/DSC) with a linear heating rate of 10 °C min^−1^ under nitrogen atmosphere, and their TG/DSC curves are shown in Figure 4. According to the TG curve of complex **1**, it undergoes a main weight loss (40.4%) in the temperature range 300–350 °C, which is attributed to the decomposition of the coordination framework. Meanwhile, the DSC curve further showed that these is only one intense exothermic peak with a peak temperature of 323.0 °C, which corresponds to its decomposition temperature. In addition, complex **2** also undergoes significant weight loss (49.8%) in the temperature range 290–370 °C, which is attributed to the decomposition of the coordination framework. There is only one exothermic process with a peak temperature of 333.3 °C in its DSC curve (Figure 4). These complexes are among very few energetic materials that show thermal stability above 300 °C [29,30,31]. Furthermore, complex **1** is more thermally stable than nearly all energetic salts and cocrystals of DNBT reported at present [11,32]. The decomposition temperature of complex **2** (333.3 °C) is also the highest among those of all reported high-energy MOFs with 1D chain structures [33]. The high thermal stabilities of these complexes are presumably caused by strong multiple intermolecular interactions such as hydrogen-bonding and π-π stacking.

Besides their high thermal stabilities, the two complexes also possess high densities. The densities of the complexes are 1.93 g cm^−3^ for complex **1** and 1.96 g cm^−3^ for complex **2**, which are higher than that of the parent monomer (DNBT, ρ = 1.90 g cm^−3^) [11]. With the introduction of N–O bonds, the density of the MOFs increases distinctly. It is worth noting that the two complexes also show higher crystal densities than those of most known copper-based MOFs such as {[Cu(ATZ)(ClO_4_)_2_]_n_, ρ = 1.40 g cm^−3^, ATZ = 4-amino-1,2,4-triazole} [34]; {[Cu(Pn)(N_3_)_2_]_n_, ρ = 1.76 g cm^−3^, Pn = 1,2-diaminopropane} [17]; {[Cu_2_(En)_2_(N_3_)_4_]_n_, ρ = 1.93 g cm^−3^, En = ethylenediamine} [35], {[Cu(ATRZ)_3_(NO_3_)_2_]_n,_ ρ = 1.68 g cm^−3^} [20] and {[Cu(Htztr)_2_(H_2_O)_2_]_n,_ ρ = 1.89 g cm^−3^, Htztr^−^ = 3-(1*H*-tetrazol-5-yl)-1*H*–triazole} [36]. It is possible that these complexes contain polynitro ligands, which result in their high densities. In addition, the complexes also have a high nitrogen content: 52.4% and 44.8%, respectively, for complexes **1** and **2**. Thus, they can not only release a large amount of energy, but solid waste containing harmful components is reduced during detonation.

Sensitivity to external stimuli, such as electrostatic discharge, friction, and impact, is important for safe handling and transportation of explosive materials. The impact sensitivity (IS) and friction sensitivity (FS) measurements of complexes **1** and **2** were performed using a standard BAM drop hammer and a BAM friction tester, respectively. The results showed that the complexes exhibit relatively low sensitivities towards impact and friction (Table 1). In particular, complex **1** is insensitive to impact and friction (IS > 40 J and FS > 360 N), and the values are lower than those of DNBT (IS = 10 J and FS = 360 N) [11], CHP (IS = 5 J) [12], CHHP (IS = 8 J) [37].

According to our developed method [38,39], the detonation properties {e.g., detonation velocity (D) and detonation pressure (P)} of the energetic MOFs were calculated using the experimentally determined (back-calculated from Δ_c_*U*) enthalpy of formation (Δ*_f_H°*), and the crystal densities. The constant-volume combustion energies (Δ*_c_U*) of the complexes were measured by an oxygen bomb calorimeter. The enthalpy of combustion (Δ*_c_H°*) was calculated from Δ*_c_U*, and correction for the change in the gas volume during combustion was included (Scheme 1, Equation (1)). The standard enthalpies of formation of complexes **1** and **2** were back-calculated from the heats of combustion on the basis of combustion equations (Scheme 1, Equations (2) and (3)), Hess’ law (Scheme 1, Equations (4) and (5)), and the known standard heats of formation of copper oxide (−157.3 kg mol^−1^), water (−285.8 kg mol^−1^), and carbon dioxide (−393.51 kg mol^−1^) [40]. The calculated Δ*_f_H°* values of complexes **1** and **2** are 1461 and 843.6 kJ mol^−1^, respectively. We used the EXPLO5 computer code (version 6.01) to calculate their detonation velocity (D) and detonation pressure (P). For complex **2**, P = 27.62 GPa and D = 7.86 km s^−1^, which are better than the same values for TNT [12], CHHP, and ZHHP, and many of known energetic MOFs. The detonation properties of some energetic MOFs and energetic complexes are listed in Table 1.

## 3. Experimental Materials and Methods

### 3.1. Chemical and Materials

Cu(NO_3_)_2_·5H_2_O, NaNO_2_ and oxalic acid were purchased from the Aladdin corporation and used without further purification, Oxone was purchased from Shanghai Alfa aesar Co. Ltd. (Shanghai, China). Aminoguanidine bicarbonate was purchased from Shanghai Macklin Biochemical Co. Ltd. (Shanghai, China). All chemicals and reagents were of analytical grade, and were used as received without further purification. Deionized water was used throughout this work.

### 3.2. Preparation of Ligands

4,4′-Azo-1,2,4-triazole (ATRZ) was prepared according to our previous work [41]. DNBT was prepared according to the procedures described in the literature [42]. In a typical synthesis of DNBTO, DNBT (1.0 g, 4.4 mmol) was dissolved in a solution of water (25 mL) and potassium acetate (5.0 g, 0.051 mol) and heated to 40 °C. Oxone (16.6. g, 27 mmol) was added portion wise within 2 h, and the pH was meanwhile carefully adjusted to 4–5 by dropwise addition of potassium acetate (38.0 g, 0.38 mol) in water (50 mL). The mixture was subsequently stirred at 40 °C for 24 h. The solution was acidified with sulfuric acid and extracted with ethyl acetate. The combined organic phases were dried over magnesium sulfate, and the solvent was evaporated in vacuum.^15^N NMR (DMSO-d_6_): δ (ppm) = 240.66, 262.70, 290.66, 353.59. ^13^C.NMR (DMSO-d_6_): δ (ppm) = 155.216(C–NO_2_), 134.66 (C–C). IR: (KBr pellets, cm^−1^) = 3459, 3413, 1659, 1556, 1462, 1412, 1315, 1045, 840, 746, 690. Ammonia (gaseous) was led through a solution of DNBTO in ethanol. The precipitate was collected by filtration to give ammonium DNBTO.

### 3.3. Synthesis of the Energetic Metal Organic Framework

The copper complex, [Cu(DNBT)(ATRZ)3]_n_ (**1**) was synthesized with a hydrothermal method; copper dinitrate pentahydrate was reacted with ATRZ and an ammonium salt of DNBT [30] in water. ATRZ (0.05 g, 0.3 mmol) was suspended in 10 mL deionized water, and stirred at room temperature until the solution was clear. Ammonium salt of DNBT (0.238 g, 0.9 mmol) and a few drops nitric acid were added. A solution of copper dinitrate pentahydrate (0.22 g, 0.9 mmol) in 20 mL water was added at room temperature and held at this temperature for 48 h, after which dark-blue bulk crystals were acquired. The solid was collected by filtration, washed with deionized water, and dried in air for 30 min. Yield: 65% based on Cu. Elemental analysis (%) calculated for C_10_H_6_CuN_20_O_4_ (M = 533.89): C, 22.47; H, 1.12; N, 52.43. Found: C, 22.38; H, 1.10; N, 51.89. IR (KBr pellets, cm^−1^): 3143, 3078, 1540, 1396, 1303, 1183, 1049, 831, 613, 423.

The synthetic method of complex **2** is same as the way of complex **1**, using ATRZ (0.05 g, 0.3 mmol), ammonium salt of DNBTO(0.267 g, 0.9 mmol) and Cu(NO_3_)_2_·5H_2_O (0.22 g, 0.9 mmol). Yield: 60% based on Cu. Elemental analysis (%) calculated for C_8_H_8_CuN_16_O_6.77_ (M = 500.16): C, 19.20; H, 1.61; N, 44.79. Found: C, 18.83; H, 1.47; N, 46.54. IR (KBr pellets, cm^−1^): 3169, 3102, 1629, 1544, 1506, 1465, 1396, 1375, 1307, 1183, 1036, 837, 765, 620, 548.

### 3.4. Measurement of Solid-State Ultraviolet Absorption

Ultraviolet absorption was tested on a UV-2600 220v CH ultraviolet spectrophotometry from Beijing Shimadzu Co. Ltd. (Beijing, China) (attaching diffuse reflection measurement device: integrating sphere). Instrument parameters: high-speed scanning rate and slit width is 1.

### 3.5. Measurement of Temperature Using Differential Scanning Calorimetry Measurement/Thermogravimetric

To determine the thermal stability of the described MOFs, a TG-DSC Q2000 differential scanning calorimeter was used. About 1.5 mg of sample was used and the temperature was programmed to 600 °C (873 K) at the rate of 10 °C min^−1^ in 60 mL min^−1^ N_2_ flow.

### 3.6. Measurement of Sensitivity

To determine the thermal stability of the described MOFs, a type 12 tooling according to the “up and down” method (Bruceton method). A 2.5 kg weight was dropped from a set height onto a 20 mg sample placed on 150 grit garnet sandpaper. Each subsequent test was made at the next lower height if explosion occurred and at the next higher height if no explosion happened. 50 drops were made from different heights, and an explosion or non-explosion was recorded to determine the results. RDX was considered as a reference compound, and the impact sensitivity of RDX is 7.4 J [43]. The friction sensitivity was tested on a FSKM-10 BAM friction apparatus. RDX was also used as a reference compound, and its friction sensitivity is 110 N [43].

### 3.7. Measurement of Single-Crystal X-ray Diffraction

The crystal structure was determined by a Rigaku RAXIS IP diffractometer and SHELXTL crystallographic software package of molecular structure. Single crystals of [Cu(DNBT)(ATRZ)_3_]_n_ (**1**) and [Cu(DNBTO)(ATRZ)_2_(H_2_O)_2_]_n_ (**2**) were mounted on a Rigaku RAXIS RAPID IP diffractometer equipped with a graphite-monochromatized MoKα radiation (λ = 0.71073 Å). Data were collected by the ω scan technique.

## 4. Conclusions

Two high-density energetic MOFs based on polynitro heterocyclic DNBT and DNBTO ligands were successfully synthesized. Their structures were characterized by FT-IR spectroscopy, elemental analysis, ultraviolet-visible (UV) absorption spectrophotometry, thermal analysis, and single-crystal X-ray diffraction. The results showed that complex **1** adopts a 2D porous framework and possesses the most stable chair conformations (like cyclohexane), whereas complex **2** adopts a 1D polymeric structure. Moreover, the complexes possess high thermal stabilities (decomposition temperatures of 323 °C for complex **1** and 333.3 °C for complex **2**) and high densities (ρ = 1.93 g cm^−3^ for complex **1** and ρ = 1.96 g cm^−3^ for complex **2**) due to their containing many nitro groups and N–O bonds. The complexes also exhibit relatively low sensitivities towards impact and friction. In particular, complex **1** is insensitive to impact and friction (IS > 40 J and FS > 360 N). Thus, we anticipate that the two complexes would be potential high-energy density materials.

## Figures and Tables

**Scheme 1:** Combustion reactions of energetic MOFs, and Hess’ Law for these combustion reactions.

∆*_C_H°* = ∆_C_*U* + ∆nRT
[∆n = ∆n_i_ (products, g) − ∆n_j_ (reactants, g), ∆n_i_ or ∆n_j_ is the total molar amount of gases in the products or reactnts] (1)

C_10_H_6_CuN_20_O_4_ (s) + 10 O_2_ (g) → CuO (s) + 10CO_2_ (g) + 3H_2_O (l) + 10N_2_ (g) (2) MOF(DNBT) (2)

C_8_H_8_CuN_16_O_6.77_ (s) + 7.115 O_2_ (g) → CuO (s) + 8CO_2_ (g) + 4H_2_O (l) + 8N_2_ (g) (3) MOF(DNBTO) (3)

∆*_f_ H°*{MOF(DNBT), s} = ∆*_f_ H°*(CuO, s) + 10∆*_f_ H°*(CO_2_, g) + 3∆*_f_ H°*(H_2_O, l) − ∆*_C_H°*{MOF(DNBT), s} (4)

∆*_f_ H°*{MOF(DNBTO), s} = ∆*_f_ H°*(CuO, s) + 8∆*_f_ H°*(CO_2_, g) + 4∆*_f_ H°*(H_2_O, l ) − ∆*_C_H°*{MOF(DNBTO), s} (5)

**Table 1:** Physiochemical properties of **1**, **2** and previously reported MOF based on copper ions.

Compound	T_dec_^a^	ρ ^b^	N ^c^	Ω_co_^d^	IS^e^	FS ^f^	−Q ^g^	P ^h^	D ^i^
**1**	323.2	1.93	52.44	−26.97	>40	>360	4346.2	25.27	7.7
**2**	333.3	1.96	44.79	−16.73	18	360	4476	27.62	7.86
[Cu(ATZ)(ClO_4_)_2_]_n_ [34]	>250	1.40	32.66	−13.35	1	8.8	-	-	6.5
[Cu(Pn)(N_3_)_2_]_n_ [17]	215.7	1.76	50.54	−57.73	2.55	-	-	-	-
[Cu_2_(En)_2_(N_3_)_4_]_n_ [35]	201.8	1.93	53.95	−46.22	7.84	-	-	-	-
[Cu(ATRZ)_3_(NO_3_)_2_]_n_ [20]	243	1.68	53.35	−28.24	22.5	112	4388	35.68	9.16
[Cu(Htztr)_2_(H_2_O)_2_]_n_ [36]	345	1.89	52.72	−34.43	>40	>360	-	30.57	8.18
RDX [12]	210	1.8	37.84	0	7.5	120	1258.3	33.92	8.6
TNT^`^ [12]	244	1.65	18.50	−24.7	15	353	852	20.50	7.178

^a^ Decomposition temperature (°C); ^b^ Density from X-ray diffraction analysis (g cm^−3^); ^c^ Nitrogen content (%); ^d^ Oxygen balance based on CO (%); ^e^ Impact sensitivity (N); ^f^ Friction sensitivity (J); ^g^ Heat of detonation (kJ kg^−1^); ^h^ Detonation pressure (GPa); ^i^ Detonation velocity (km s^−1^). ATZ = 4-amino-1,2,4-triazole; Pn = 1,2-diaminopropane; En = ethylenediamine; ATRZ = 4,4′-azo-1,2,4-triazole; Htztr = 3-(1*H*-tetrazol-5-yl)-1*H*–triazole; RDX = 1,3,5-trinitro-1,3,5-triazacyclohexane; TNT = 2,4,6-trinitrotoluene.

## Supplementary Materials

The following are available online. Figure S1: Coordination structure of complex **1** (left) and complex **2** (right); Figure S2: FTIR spectrum of complex **1** (left) and complex **2** (right); Figure S3: Experimental PXRD pattern of complexes **1** and **2**; Table S1: Crystal data and structure refinement details for **1** and **2**; Table S2: Selected bond lengths and bond angles for **1**; Table S3: Selected bond lengths and bond angles for **2** and Table S4: Selected hydrogen-bond lengths for **2**.
